# Mineral and heavy metal content in canned tuna: Implications for veterinary public health and consumer safety

**DOI:** 10.1007/s11259-026-11228-x

**Published:** 2026-04-29

**Authors:** Nermin Rüstemli, Rabia Mehtap Tuncay

**Affiliations:** 1 Health Sciences Institute, Van Yüzüncü Yıl University, Van, Türkiye 65080 Turkey; 2 Faculty of Veterinary Medicine, Department of Food Hygiene and Technology, Van Yüzüncü Yıl University, Van, Türkiye 65080 Turkey

**Keywords:** Canned tuna Trace elements, Mercury, Arsenic, Health risk assessment, Target hazard quotient, Seafood contamination, Turkish market

## Abstract

This study evaluated the concentrations of minerals and heavy metals in canned tuna products available on the Turkish market, with a particular focus on the influence of packaging medium (water, oil, and sauce) on elemental distribution. A total of 105 canned tuna samples were collected from Van Province, Türkiye, comprising equal groups (*n* = 35) of water-, oil-, and sauce-packed products. Following microwave-assisted acid digestion, concentrations of 15 elements (magnesium (Mg), aluminum (Al), phosphorus (P), potassium (K), calcium (Ca), chromium (Cr), manganese (Mn), iron (Fe), copper (Cu), zinc (Zn), arsenic (As), selenium (Se), tin (Sn), mercury (Hg), and lead (Pb)) were determined using inductively coupled plasma mass spectrometry (ICP-MS). Method accuracy was verified using DORM-4 Fish Protein certified reference material, yielding recoveries between 95% and 102% for all analyzed elements. Oil-packed tuna exhibited significantly higher concentrations of most elements compared to water- and sauce-packed samples (*p* < 0.05), with 2–4-fold differences observed for Mg, P, Fe, and Hg. Mercury concentrations ranged from 0.06 to 2.68 mg/kg, with an overall mean of 0.41 mg/kg, values exceeded the Turkish Food Codex limit (1.0 mg/kg) in 5.71% of samples, predominantly in oil-packed products (mean: 0.62 mg/kg). Arsenic concentrations ranged from 0.31 to 7.08 mg/kg (overall mean: 1.49 mg/kg) and were frequently detected across all packaging types (74–100% of samples). All samples complied with regulatory limits for lead and tin. Health risk assessment based on estimated daily intake and target hazard quotient indicated a potential non-carcinogenic risk associated with arsenic exposure, while mercury exposure approached the risk threshold, particularly for oil-packed tuna. The ICP-MS method, assessed through certified reference material and quality control procedures, demonstrated adequate sensitivity and reliability for elemental analysis of canned tuna products and supports food safety assessment in the Turkish seafood market.

## Introduction

Fish and seafood products constitute a vital component of the global diet, providing high-quality protein, omega-3 polyunsaturated fatty acids (particularly eicosapentaenoic acid (EPA) and docosahexaenoic acid (DHA), and essential micronutrients such as iodine, selenium, vitamin D, and vitamin B12 (FAO 2020; Mozaffarian and Rimm 2006). For example, a 100 g serving of tuna provides approximately 25–30 g of protein, covering more than 50% of the adult daily requirement, and supplies significant amounts of selenium and vitamin B12 that are otherwise difficult to obtain from plant-based diets (EFSA 2006; Baysal 2012). Additionally, omega-3 fatty acids derived from seafood are widely recognised for their cardioprotective, anti-inflammatory, and neuroprotective effects (Mozaffarian and Rimm 2006). Canned tuna, in particular, represents one of the most widely consumed seafood products worldwide, with global tuna production exceeding 4.5 million metric tons per year (Herpandi et al. 2011; FAO 2023). These products are valued for their affordability, convenience, extended shelf life, and rich nutritional profiles. Essential minerals such as magnesium (Mg), calcium (Ca), potassium (K), phosphorus (P), iron (Fe), zinc (Zn), copper (Cu), and selenium (Se) play important roles in metabolism, immune function, bone health, and enzymatic activity (Uysal eand Atalay 2007; Duran et al. 2014; Baysal 2012).

However, the bioaccumulation of toxic heavy metals in marine fish, particularly mercury (Hg), lead (Pb), arsenic (As), and aluminum (Al) poses significant public health concerns. These contaminants originate from industrial discharge, agricultural runoff, and atmospheric deposition, accumulate through aquatic food chains, and reach elevated concentrations in predatory species such as tuna (Tsai et al. 2003; Mumford et al. 2007). Mercury, especially in its methylmercury form, is neurotoxic and has been associated with cognitive impairment, cardiovascular disease, and immunosuppression (Voegborlo et al. 1999; Khansari et al. 2005). Chronic arsenic exposure increases cancer risk and causes cardiovascular complications (Marshall et al. 2007; McCarty et al. 2007), while aluminum accumulation may contribute to neurodegenerative disorders (Çelik 2017; Akalın 2018).

In Türkiye, per capita fish consumption averages approximately 6.2 kg per year, remaining well below the global average of 20.3 kg (TSI 2020; FAO 2020). Despite being surrounded by seas on three sides, per capita consumption has remained relatively stagnant over the past decade. Van Province, located in eastern Türkiye with a population exceeding 1.1 million, represents a geographically and economically distinct region where border proximity influences food import patterns and market diversity. However, systematic surveillance data on the mineral composition and heavy metal contamination of canned tuna products from this region remain scarce.

Previous studies have investigated heavy metal levels in canned fish from various countries, including Jordan (Ababneh and Al-Momani 2013), Serbia (Novakov et al. 2017), and Brazil (De Lima et al. 2021). However, comprehensive assessments that combine essential minerals and toxic metals across different packaging media in the Turkish market remain limited.

Regulatory authorities, including the Turkish Food Codex (TFC 2011; 2017), Codex Alimentarius Commission (CAC 2025), and European Food Safety Authority (EFSA 2006), have established maximum permissible limits for heavy metals in fishery products to protect consumer health. The packaging medium (water, oil, or sauce) may influence metal retention and bioavailability, however, comparative studies addressing this factor remain underrepresented. Furthermore, the balance between nutritional benefits from essential minerals and health risks from toxic metals requires integrated risk-benefit assessment.

This study aimed to comprehensively evaluate the elemental composition of canned tuna products marketed in Van Province, Türkiye, by determining the concentrations of 15 elements (Mg, Al, P, K, Ca, Cr, Mn, Fe, Cu, Zn, As, Se, Sn, Hg, and Pb). Elemental accumulation patterns were compared across three packaging media: water, oil, and sauce. Measured concentrations were assessed for compliance with maximum permissible limits established by the Turkish Food Codex (TFC) and Codex Alimentarius Commission (CAC). Potential health risks associated with canned tuna consumption were evaluated using estimated daily intake (EDI) and target hazard quotient (THQ) approaches. The findings provide baseline data for food safety monitoring, support evidence-based regulatory decision-making, and contribute to public health risk assessment at national and international levels.

## Materials and methods

### Study design and ethical approval

This cross-sectional analytical study was conducted between September and November 2022 in Van Province, Türkiye. The study protocol was approved by the Van Yüzüncü Yıl University Non-Interventional Clinical Research Ethics Committee (Decision No. 2022/11 − 03, dated November 3, 2022).

### Sample collection and characterization

A total of 105 canned tuna samples were collected from large retail chains, local supermarkets, and online platforms across Van Province between September and November 2022. The samples represented seven domestic Turkish brands, each available in three product types (water-packed, oil-packed, and sauce-packed). Multiple production lot numbers were included for certain brands to account for batch-to-batch variability, while brand identities are not disclosed in accordance with ethical considerations. The samples comprised three equal groups (*n* = 35 each): water-packed, oil-packed, and sauce-packed tuna. Store selection was based on geographical distribution across urban and peri-urban areas to capture market diversity. Sample size was determined by power analysis (α = 0.05, power = 0.80, effect size = 0.5), requiring a minimum of 30 samples per group to detect significant differences in metal concentrations among packaging types.

Sauce-packed samples included Mediterranean-style, barbecue, mustard, jalapeño pepper, mayonnaise, sun-dried tomato, and hot sauce varieties. Oil-packed samples contained sunflower oil, olive oil, or mixed vegetable oils. Net weight ranged from 80 to 160 g per unit. To account for batch-to-batch variability, at least three different production lots per brand were included. Only commercially available products within their expiration date and with intact, undamaged packaging were selected. All samples were stored at room temperature (20–25 °C) in their original packaging until analysis.

### Sample preparation

Sample preparation followed AOAC Official Method 999.10 (AOAC 2005) with minor modifications. Water-packed tuna samples (tuna + liquid) were transferred to pre-cleaned glass beakers and homogenized using a high-speed blender (IKA T25 Digital Ultra-Turrax, Germany) for 2 min at 10,000 rpm. Approximately 10 g of the homogenized sample was weighed into pre-weighed porcelain crucibles and oven-dried at 105 ± 2 °C until constant weight was achieved (typically 18–24 h).

Oil-packed and sauce-packed samples were freeze-dried (Sanyo MDF U32865, Japan) at −40 °C for 18 h to facilitate homogenization and improve digestion efficiency. Freeze-dried samples were ground into fine powder using a ceramic mortar and pestle to prevent metal contamination. Homogenized powders were stored in acid-washed polypropylene containers at −20 °C until digestion.

### Microwave-assisted acid digestion

Sample digestion was performed using an ETHOS EASY microwave digestion system (Milestone, Italy) with 65% suprapur-grade nitric acid (HNO₃, Merck 100456, Germany) and ultrapure Type I water (18.2 MΩ·cm, Milli-Q system). Approximately 200 ± 5 mg of dried or freeze-dried sample was accurately weighed into Teflon (TFM) digestion vessels. Subsequently, 7 mL of concentrated HNO₃ and 1 mL of ultrapure water were added. Following a 30 min pre-digestion step at room temperature, microwave-assisted digestion was performed using a three-step temperature program up to 200 °C.

Cooled digests were quantitatively transferred into acid-washed 15-mL polypropylene tubes, and the final volume was adjusted to 15 mL with ultrapure water. Digestates were stored at 4 °C and analyzed by ICP–MS within 48 h.

### Quality control

Quality control procedures included procedural blanks (three per batch of 10–12 samples), certified reference material (DORM-4 Fish Protein, NRCC) analyzed in triplicate with each batch, and matrix spike recovery tests performed on approximately 10% of randomly selected samples (Willie et al. 2012). Duplicate analyses of 10% of samples were conducted to assess method precision, digestion efficiency, and matrix effects.

### Inductively coupled plasma mass spectrometry (ICP–MS) analysis

Elemental analysis was conducted using an Agilent 7800 ICP–MS (Agilent Technologies, USA) at the Central Research Laboratory of Bayburt University, Türkiye. Samples were introduced via a MicroMist concentric nebulizer coupled with a Scott-type double-pass spray chamber cooled to 2 °C. Analyses were performed in helium collision mode or no-gas mode to reduce polyatomic interferences. Measurements were performed in triplicate with internal standards (⁷²Ge, ¹¹⁵In, and ²⁰⁹Bi; 10 µg/L) added online to compensate for instrumental drift, matrix effects, and signal fluctuations.

### Calibration and method performance

Calibration standards were prepared daily by serial dilution of 1000 mg/L certified stock solutions (Merck CertiPUR^®^, Germany) in 2% (v/v) HNO₃, which was also used for matrix matching with digested samples. Element-specific calibration ranges were applied, and multi-point external calibration curves (six points plus blank) showed excellent linearity for all elements (R²>0.999). Calibration accuracy was verified using second-source standards and mid-range check standards analyzed every ten samples, with acceptable recoveries between 90% and 110%. Limits of detection (LOD) and quantification (LOQ) were calculated as three and ten times the standard deviation of procedural blanks, respectively.

### Quality assurance and quality control (QA/QC)

Method detection limits (LOD) and quantification limits (LOQ) were calculated as three and ten times the standard deviation of procedural blanks (*n* = 10), respectively. Concentrations below the LOQ were reported as < LOQ, and values below the LOD were assigned as LOD/2 for statistical analyses. All LOD values were at least one order of magnitude lower than the minimum expected sample concentrations, confirming adequate method sensitivity for regulatory compliance assessment.

Method accuracy was assessed using DORM-4 Fish Protein Certified Reference Material (NRCC), selected due to its close matrix similarity to fish products and its certified concentrations for trace and toxic elements relevant to food safety. The CRM was digested and analyzed under identical conditions as the study samples, and recovery values for all analyzed elements were within acceptable ranges (95–102%), confirming the accuracy and reliability of the analytical method (Willie et al. 2012).

### Health risk assessment

Potential non-carcinogenic health risks associated with heavy metal exposure through canned tuna consumption were assessed using the estimated daily intake (EDI) and target hazard quotient (THQ) approaches. The EDI (mg/kg body weight/day) was calculated using the equation:

$$\:EDI=\frac{C\times\:IR}{BW}$$

where C is the mean metal concentration (mg/kg, wet weight), IR is the daily ingestion rate of canned tuna (0.033 kg/day), and BW is the average adult body weight (70 kg).

The THQ was calculated as the ratio of EDI to the oral reference dose (RfD) established by the United States Environmental Protection Agency (EPA) (Chien et al. 2002):

$$\:THQ=\frac{EDI}{RfD}$$

A THQ value below 1 indicates no significant health risk, whereas THQ ≥ 1 suggests a potential non-carcinogenic health risk.

### Statistical analysis

The Shapiro–Wilk test was applied to assess the normality of data distribution. After confirming normal distribution, one-way analysis of variance (ANOVA) was performed to evaluate differences among packaging media groups. Duncan’s multiple range test was used for post-hoc pairwise comparisons. Statistical significance was set at *p* < 0.05. All statistical analyses were performed using IBM SPSS Statistics Version 25.

## Results

Heavy metal and mineral concentrations were determined in 105 canned tuna samples comprising three preservation media: sauce (*n* = 35), oil (*n* = 35), and water (*n* = 35). A total of 15 elements (Mg, Al, P, K, Ca, Cr, Mn, Fe, Cu, Zn, As, Se, Sn, Hg, and Pb) were analyzed. Each sample was analyzed in triplicate, and mean values were used for statistical analysis.

The Shapiro–Wilk test confirmed the normal distribution of the data (*p* > 0.05). One-way ANOVA revealed significant differences among the three preservation media groups (*p* < 0.05). Duncan’s multiple range test was performed for post-hoc pairwise comparisons (Tables 1, 2 and 3).

**Table 1:** Concentrations of essential macroelements in canned tuna samples by preservation medium (mg/kg wet weight)

Element	Preservation Medium	Mean ± SD	Range (Min–Max)	Statistical Group^a^
**Magnesium (Mg)**	Sauce	549.78 ± 281.45	230.21–1330.62	b
	Oil	1787.52 ± 531.27	1078.55–3640.31	a
	Water	524.28 ± 147.96	283.25–899.02	b
**Aluminum (Al)**	Sauce	13.99 ± 5.42	9.57–33.10	a
	Oil	18.67 ± 6.12	10.83–33.94	a
	Water	13.65 ± 2.97	9.31–19.67	a
**Phosphorus (P)**	Sauce	2253.64 ± 349.42	1715.90–3105.56	b
	Oil	9594.28 ± 1678.35	6867.76–13562.61	a
	Water	2218.94 ± 295.46	1735.68–2745.68	b
**Potassium (K)**	Sauce	2398.45 ± 402.18	1698.63–3224.63	b
	Oil	13048.38 ± 3638.74	9368.36–20845.60	a
	Water	2387.46 ± 485.73	1600.93–3447.91	b
**Calcium (Ca)**	Sauce	128.95 ± 62.48	20.29–281.18	b
	Oil	207.08 ± 104.82	64.70–561.21	a
	Water	102.58 ± 48.93	37.19–240.21	b
**Iron (Fe)**	Sauce	22.08 ± 10.93	12.13–70.52	b
	Oil	89.97 ± 32.79	43.15–186.71	a
	Water	20.35 ± 6.95	9.71–32.64	b
**Manganese (Mn)**	Sauce	0.73 ± 0.39	0.28–2.08	ab
	Oil	0.86 ± 0.27	0.54–1.30	a
	Water	0.49 ± 0.24	0.16–1.12	b
**Chromium (Cr)**	Sauce	0.47 ± 0.10	0.26–0.89	b
	Oil	0.60 ± 0.34	0.37–2.10	a
	Water	0.48 ± 0.09	0.36–0.67	b

Values are presented as mean ± standard deviation (*n* = 35 per group). ^a^Different superscript letters (a, b, ab) indicate significant differences among preservation media (Duncan’s multiple range test, *p* < 0.05)

**Table 2:** Concentrations of trace elements in canned tuna samples by preservation medium (mg/kg wet weight)

Element	Preservation Medium	Mean ± SD	Range (Min–Max)	Statistical Group^a^
**Cobalt (Co)**	Sauce	0.034 ± 0.047	0.009–0.269	a
	Oil	0.026 ± 0.010	0.011–0.050	a
	Water	0.035 ± 0.016	0.019–0.092	a
**Nickel (Ni)**	Sauce	0.379 ± 0.145	0.148–0.989	a
	Oil	0.393 ± 0.238	0.077–1.053	a
	Water	0.423 ± 0.115	0.120–0.667	a
**Copper (Cu)**	Sauce	1.040 ± 0.263	0.493–1.546	b
	Oil	2.388 ± 0.647	1.487–3.887	a
	Water	0.811 ± 0.538	0.000–2.078	b
**Zinc (Zn)**	Sauce	46.82 ± 28.35	8.22–117.12	a
	Oil	67.33 ± 19.78	26.07–95.88	a
	Water	17.84 ± 18.42	0.00–56.00	b
**Arsenic (As)**	Sauce	0.782 ± 0.329	0.310–1.867	b
	Oil	3.039 ± 1.525	0.790–7.083	a
	Water	0.641 ± 0.177	0.370–0.990	b
**Selenium (Se)**	Sauce	0.833 ± 0.254	0.520–1.350	b
	Oil	3.646 ± 0.627	2.755–5.036	a
	Water	0.720 ± 0.194	0.120–1.000	b
**Strontium (Sr)**	Sauce	8.174 ± 4.437	3.809–21.923	b
	Oil	12.348 ± 6.516	5.116–34.100	a
	Water	7.087 ± 3.473	3.339–16.422	b

Values are presented as mean ± standard deviation (*n* = 35 per group). ^a^Different superscript letters (a, b) indicate significant differences among preservation media (Duncan’s multiple range test, *p* < 0.05)

**Table 3:** Concentrations of potentially toxic elements in canned tuna samples by preservation medium and comparison with regulatory limits (mg/kg wet weight)

Element	Preservation Medium	Mean ± SD	Range (Min–Max)	Statistical Group^a^	EU/WHO Limit^*^	TFC Limit^**^
**Tin (Sn)**	Sauce	39.52 ± 3.26	34.00–44.79	b	200	200
	Oil	59.41 ± 20.83	38.21–115.87	a		
	Water	37.07 ± 2.92	32.64–43.13	b		
**Barium (Ba)**	Sauce	4.309 ± 1.270	3.224–8.413	a	–	–
	Oil	5.348 ± 1.620	3.362–9.197	a		
	Water	4.491 ± 1.550	3.826–11.100	a		
**Mercury (Hg)**	Sauce	0.380 ± 0.283	0.080–1.280	ab	0.50–1.00	1.00
	Oil	0.617 ± 0.402	0.190–2.680	a		
	Water	0.153 ± 0.074	0.060–0.380	b		
**Lead (Pb)**	Sauce	0.094 ± 0.041	0.050–0.270	a	0.30	0.30
	Oil	0.104 ± 0.055	0.040–0.252	a		
	Water	0.053 ± 0.026	0.000–0.100	b		

Values are presented as mean ± standard deviation (*n* = 35 per group). ^a^Different superscript letters (a, b, ab) indicate significant differences among preservation media (Duncan’s multiple range test, *p* < 0.05). ^*^EU Regulation 1881/2006 and WHO guidelines for fish and fishery products. ^**^Turkish Food Codex Contaminants Regulation (Official Gazette No: 32360, Date: 05.11.2023)

### Elemental concentrations by preservation medium

**Fig. 1:**
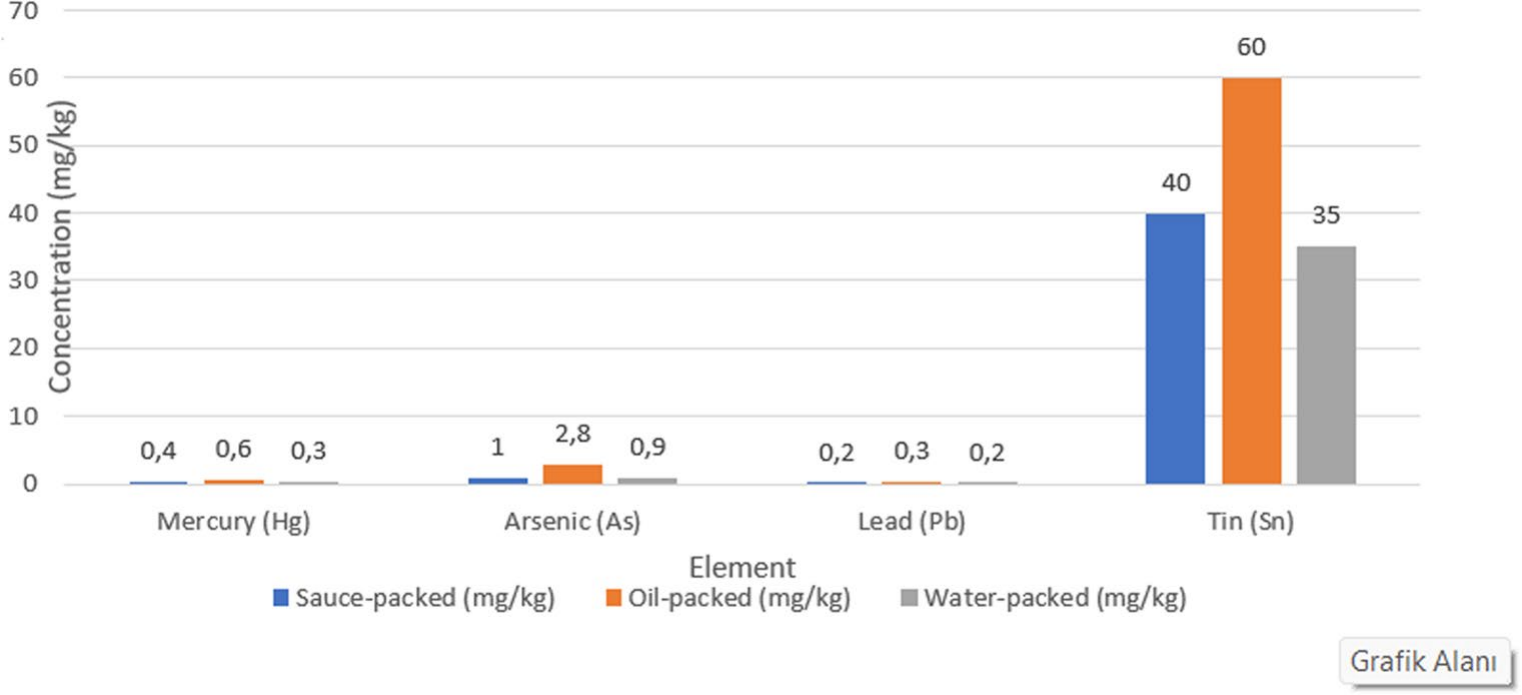
Comparison of essential element concentrations in canned tuna according to preservation medium. Regulatory limits - Mercury: 1.0 mg/kg (TFC), Lead: 0.30 mg/kg, Tin: 200 mg/kg. Values represent mean concentrations (n=35 per group)

**Fig. 2:**
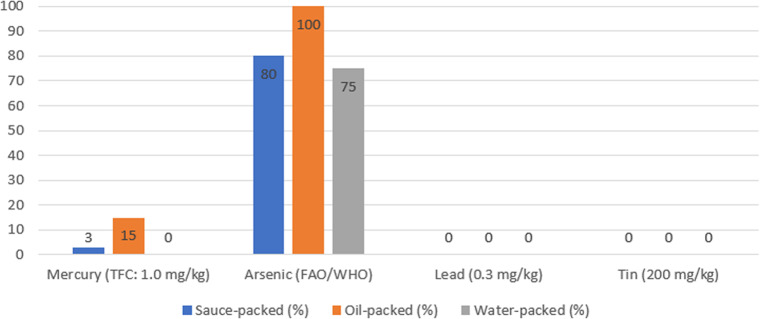
Percentage of Samples Exceeding Regulatory Limits. Percentage values are displayed above each bar for each packaging medium

**Fig. 3:**
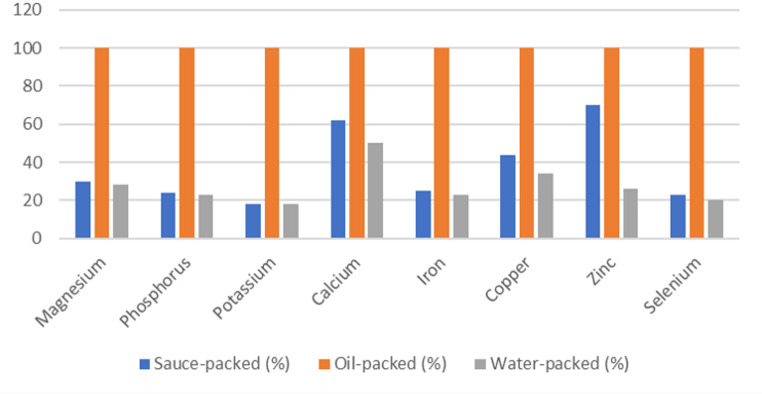
Comparison of essential element concentrations in canned tuna according to packaging medium. Mean values (mg/kg wet weight, n=35 per group) are displayed above each bar

Element concentrations varied significantly among preservation media (*p* < 0.05). Oil-packed tuna exhibited the highest concentrations for most of the analyzed elements, including Mg (1787.52 ± 531.27 mg/kg), P (9594.28 ± 1678.35 mg/kg), K (13048.38 ± 3638.74 mg/kg), Fe (89.97 ± 32.79 mg/kg), Cu (2.388 ± 0.647 mg/kg), As (3.039 ± 1.525 mg/kg), Se (3.646 ± 0.627 mg/kg), and Hg (0.617 ± 0.402 mg/kg). Water-packed samples generally showed the lowest metal concentrations, while sauce-packed samples demonstrated intermediate levels (Figs. 1, 2 and 3).

Oil-packed tuna consistently showed the highest concentrations across essential elements, with values 1.5–4 times higher than water-packed alternatives. For instance, magnesium concentrations were approximately 3.4-fold higher in oil-packed (1787.52 mg/kg) than water-packed samples (524.28 mg/kg), phosphorus concentrations were 4.3-fold higher (9594.28 vs. 2218.94 mg/kg), iron concentrations were 4.4-fold higher (89.97 vs. 20.35 mg/kg), and mercury concentrations were 4.0-fold higher (0.617 vs. 0.153 mg/kg). This trend was observed for both nutritionally beneficial and potentially toxic elements essential elements. Essential macroelement concentrations are presented in Table 1, trace elements in Table 2, and potentially toxic elements with regulatory comparisons in Table 3.

### Regulatory compliance

Regarding regulatory compliance, mercury levels exceeded the Turkish Food Codex (2023) limit (1.0 mg/kg) in six samples (5.71%): one sauce-packed sample (2.86%) with a mercury concentration of 1.28 mg/kg, and five oil-packed samples (14.29%) with individual concentrations of 1.05, 1.11, 1.20, 1.35, and 2.68 mg/kg, all exceeding the Turkish Food Codex (2023) limit of 1.0 mg/kg. Three of these samples (1.35 and 2.68 mg/kg in oil-packed, 1.28 mg/kg in sauce-packed) also exceeded the Codex Alimentarius Commission limit of 1.2 mg/kg. Three samples (2.86%) exceeded the Codex Alimentarius Commission limit (2024) (1.2 mg/kg). All samples complied with regulatory limits for lead (0.30 mg/kg) and tin (200 mg/kg). However, arsenic concentrations exceeded FAO/WHO guideline values in 80% of oil-packed, 80% of sauce-packed, and 74.29% of water-packed samples (Tables 1, 2 and 3).

### Health risk assessment

Estimated daily intake (EDI) and target hazard quotient (THQ) values were calculated for mercury (Hg), arsenic (As), and lead (Pb) based on the highest mean concentrations observed in oil-packed tuna samples to represent a worst-case exposure scenario. The calculated EDI values were 0.000291 mg/kg body weight/day for Hg, 0.001433 mg/kg body weight/day for As, and 0.000024 mg/kg body weight/day for Pb.

Corresponding THQ values were 0.97 for Hg, 4.78 for As, and 0.007 for Pb. THQ values indicated a potential non-carcinogenic health risk associated with arsenic exposure (THQ > 1), while mercury exposure approached the risk threshold. Lead exposure did not pose a significant health risk (THQ < 1) (Table 4).

**Table 4:** Estimated daily intake (EDI) and target hazard quotient (THQ) values for selected heavy metals based on oil-packed canned tuna consumption

Metal	EDI (mg/kg bw/day)	RfD (mg/kg bw/day)	THQ
Hg	0.000291	0.0003	0.97
As	0.001433	0.0003	4.78
Pb	0.000024	0.0035	0.007

## Discussion

This comprehensive analysis of 105 canned tuna samples from the Turkish market demonstrated significant variations in macro- and microelement concentrations across three preservation media: sauce, oil, and water. The findings revealed that oil-packed tuna consistently exhibited the highest accumulation of both essential minerals (Mg, P, K, Ca, Fe, Cu, Zn, Se) and potentially toxic elements (As, Hg, Sn), while water-packed samples generally displayed the lowest concentrations. Sauce-packed products demonstrated intermediate levels for most analyzed elements. These results have important implications for food safety, public health, and dietary exposure assessment, particularly regarding the balance between nutritional benefits and potential health risks associated with heavy metal contamination.

### Influence of packaging medium on metal accumulation

The substantially higher elemental concentrations observed in oil-packed tuna compared to sauce- and water-packed samples can be attributed to multiple interacting physicochemical factors. The lipid matrix of vegetable oils facilitates the retention and stabilization of both essential and potentially toxic elements during thermal processing and extended storage periods. This phenomenon has been consistently documented in previous investigations of oil-preserved fish products (Mol 2011; De Lima et al. 2021; Anishchenko et al. 2017). Furthermore, the oil-based medium may enhance the migration of certain metallic elements from processing equipment, tin-plated cans, and packaging materials, particularly affecting the concentrations of tin, iron, and aluminum. However, these mechanisms are likely multifactorial and involve complex interactions between the fish matrix, oil composition, processing conditions, and storage environment, warranting systematic investigation to elucidate the precise contribution of each factor.

Conversely, water-packed samples consistently exhibited the lowest metal concentrations across the majority of analyzed elements. This observation suggests that aqueous preservation media actively promote the leaching and diffusion of metallic elements from the fish tissue matrix into the surrounding liquid, which is customarily discarded prior to consumption, thereby substantially reducing dietary exposure. Sauce-packed samples demonstrated intermediate elemental levels, a pattern that likely reflects the combined effects of both aqueous and lipid components present in various sauce formulations (Mediterranean-style, barbecue, mustard, and others), along with additional contributions from spices, seasonings, and preservative additives incorporated during sauce preparation. The variations observed among studies examining canned fish products may be attributed to differences in the oils and spices utilized during production, manufacturing conditions, processing equipment specifications, storage parameters, and the geographic origin and production methods of these ingredients. It is crucial to ensure that raw materials, including oils and spices, originate from sources free from industrial contamination, environmental pollution, or proximity to highways and other potential contamination sources.

### Essential minerals: Nutritional benefits and potential health concerns

The essential elements quantified in this study include macroelements (magnesium (Mg), phosphorus (P), potassium (K), calcium (Ca) and trace elements (chromium (Cr), manganese (Mn), iron (Fe), copper (Cu), zinc (Zn), and selenium (Se). These elements are indispensable for a wide range of physiological functions, including enzyme activation, oxygen transport, antioxidant defence, skeletal integrity, and cellular energy metabolism (EFSA 2006; WHO 1996; Baysal 2012). The elements are discussed below in the order of their atomic number, following standard chemical convention, beginning with macroelements and proceeding to trace elements.

Canned tuna products demonstrated remarkably high concentrations of nutritionally essential macroelements, with particularly elevated levels observed in oil-packed varieties. Magnesium concentrations in oil-packed samples reached 1787.52 ± 531.27 mg/kg (maximum: 3640.31 mg/kg), approximately threefold higher than water-packed samples (524.28 ± 147.96 mg/kg), with 89.52% of all analyzed samples exceeding the daily reference intake of 375 mg established by TFC (2017). The mean magnesium concentration across all 105 samples was substantially higher than values reported by Anishchenko et al. (2017) for canned garfish, though lower than those documented by Hanis et al. (2024) for canned tuna from different geographic regions. While magnesium is essential for numerous physiological processes, excessive intake may lead to hypermagnesemia, a potentially serious condition particularly in individuals with compromised kidney function (Kuzu et al. 2014). Therefore, moderation in daily canned tuna consumption is advisable, especially for vulnerable populations.

Phosphorus levels exhibited similar patterns, with mean concentrations of 4685.058 ± 349.651 mg/kg across all samples and oil-packed tuna reaching 9594.28 ± 1678.35 mg/kg. Importantly, all analyzed samples (100%) exceeded the reference intake value of 700 mg established by TFC (2017) for healthy individuals aged ≥ 4 years, and all oil-packed samples surpassed the EFSA (2006) reference threshold of 3000 mg for normal healthy adults. These phosphorus concentrations were substantially higher than those reported by Anishchenko et al. (2017) in canned garfish. Although phosphorus is indispensable for skeletal integrity and cellular energy metabolism, excessive daily intake exceeding 750 mg may induce adverse gastrointestinal symptoms including diarrhea, nausea, and vomiting (EFSA 2006), underscoring the importance of balanced consumption patterns.

Potassium content varied considerably depending on packaging medium. Sauce-packed samples ranged from 1715.90 to 3105.56 mg/kg, water-packed from 1735.68 to 2745.68 mg/kg, while oil-packed tuna exhibited the highest concentrations (13048.38 ± 3638.74 mg/kg), with maximum values reaching 13562.61 mg/kg and minimum at 6867.76 mg/kg. According to TFC (2017), the recommended daily intake of potassium for healthy individuals is 2000 mg. Our findings revealed that 85.71% of sauce-based, 80% of water-based, and all oil-packed tuna samples (100%) exceeded this reference value. Furthermore, all oil-packed samples surpassed the EFSA (2006) daily requirement range of 3.1–3.5 g/day, with only 4.76% of total analyzed samples remaining within acceptable limits. These potassium concentrations were lower than those reported by Anishchenko et al. (2017) for canned garfish. Excessive potassium intake poses significant health risks, particularly in hypertensive individuals and those with chronic kidney disease, where it may precipitate significant hyperkalemia and associated complications (Aygencel 2018).

Calcium concentrations were comparatively modest, with the highest detected level at 561.21 mg/kg, remaining below the daily requirement of 800 mg specified by TFC (2017) for healthy individuals. All samples were also substantially lower than the tolerable upper intake level of 2500 mg established by EFSA (2006) for adults, pregnant women, and lactating mothers. The calcium values obtained in this study were lower than those determined by Anishchenko et al. (2017) for canned garfish and Hanis et al. (2024) for canned tuna, while De Lima et al. (2021) reported calcium levels below analytical detection limits in certain oil-packed tuna samples. These findings indicate that additional calcium-rich foods should be incorporated into the diet when canned tuna is consumed as a primary protein source to ensure adequate calcium intake.

Trace elements including chromium, manganese, iron, copper, zinc, and selenium also exhibited elevated concentrations, predominantly in oil-packed samples. Chromium concentrations averaged 0.479 ± 0.014 mg/kg in sauce-based, 0.475 ± 0.009 mg/tuna, with an overall mean of 0.735 ± 0.037 mg/kg. Only two samples (1.90%)—one sauce-based and one oil-based—fell within acceptable limits established by NRC (Jarosz-Krzemińska et al. 2020) and EFSA (2006). Excessive manganese exposure above regulatory thresholds is associated with neurotoxicity, potentially leading to significant neurological disorders (Avila et al. 2013), emphasizing the public health importance of monitoring manganese concentrations in canned seafood products.

Iron concentrations were notably elevated, with mean values of 21.993 ± 1.774 mg/kg in sauce-based, 20.647 ± 1.039 mg/kg in water-packed, and 92.224 ± 5.353 mg/kg in oil-packed tuna, yielding an overall mean of 44.955 ± 3.785 mg/kg across all samples. Importantly, 91.43% of samples exceeded the daily reference intake of 14 mg established by TFC (2017), and 31.43% surpassed the acute oral toxicity threshold of 60 mg/kg defined by EFSA (2006), which may prove lethal under certain circumstances. While iron is essential for oxygen transport and enzymatic functions, excessive intake has been associated with oxidative tissue damage, coronary heart disease, and increased cancer risk (Kar 2011). These iron levels substantially exceeded those reported in numerous previous studies (Tuzen and Soylak 2007; Ashraf et al. 2006; Mol 2011; Iwegbue 2015; Novakov et al. 2017; Anishchenko et al. 2017; De Lima et al. 2021; Hanis et al. 2024).

Copper concentrations averaged 1.044 ± 0.043 mg/kg in sauce-packed, 0.993 ± 0.098 mg/kg in water-packed, and 2.332 ± 0.011 mg/kg in oil-packed samples, with an overall mean of 1.505 ± 0.082 mg/kg. Among analyzed samples, 54.29% of sauce-based, 40% of water-based, and all oil-based tuna (100%) exceeded the daily recommended copper intake of 1 mg/day established by TFC (2017). These concentrations were generally higher than values reported by multiple investigators (Tuzen and Soylak 2007; Mol 2010, 2011; Iwegbue 2015; Anishchenko et al. 2017; Ashraf et al. 2006; Çelik and Oehlenschläger 2007; De Lima et al. 2021), though lower than certain studies (Novakov et al. 2017; Aziz et al. 2024; Al-Kazaghly et al. 2025). While copper is essential for various metabolic processes, frequent consumption may increase the risk of copper toxicity, particularly in susceptible individuals.

Zinc concentrations were measured at 45.845 ± 4.307 mg/kg in sauce-packed, 18.859 ± 3.174 mg/kg in water-packed, and 67.073 ± 3.020 mg/kg in oil-packed samples, with an overall mean of 44.413 ± 2.813 mg/kg. Among all samples, 79.05% exceeded the TFC (2017) daily zinc intake recommendation of 10 mg/day. Furthermore, 87.62% exceeded the population reference intake level for women, and 79.05% exceeded the level for men. These zinc concentrations were generally lower than those reported in the majority of comparative studies (Çelik and Oehlenschläger 2007; Herrera-Herrera et al. 2019; Ashraf et al. 2006; Tuzen and Soylak 2007; Mol 2010, 2011; Iwegbue 2015; Novakov et al. 2017; Anishchenko et al. 2017; Al Ghoul et al. 2020; De Lima et al. 2021). Regular consumption exceeding recommended levels may precipitate gastrointestinal disturbances including nausea, vomiting, and diarrhea (Plum et al. 2010).

Selenium concentrations were particularly elevated, with mean values of 0.840 ± 0.042 mg/kg in sauce-based, 0.712 ± 0.032 mg/kg in water-packed, and 3.684 ± 0.104 mg/kg in oil-packed samples, yielding an overall mean of 1.746 ± 0.140 mg/kg. All analyzed samples (100%) exceeded the daily recommended selenium intake of 55 µg established by TFC (2017). These values were lower than those reported by Tuzen and Soylak (2007) for canned tuna, sardines, and horse mackerel, but higher than findings by De Lima et al. (2021) in canned bonito and anchovy. While selenium possesses important antioxidant properties and is essential for various physiological functions, excessive dietary intake has been associated with increased risk of type 2 diabetes (Fairweather-Tait et al. 2011), necessitating careful consideration of consumption patterns.

Aluminum is not considered an essential element in human nutrition and carries no established beneficial physiological role. It was included in this study as a potentially toxic contaminant of public health significance, given its well-documented association with neurodegenerative disorders and its capacity to migrate from processing equipment and packaging materials into food matrices (Çelik 2017; Akalın 2018). Aluminum concentrations were highest in oil-packed tuna (mean: 18.67 ± 6.12 mg/kg; maximum: 33.94 mg/kg) and lowest in water-packed samples (mean: 13.65 ± 2.97 mg/kg; minimum: 9.31 mg/kg). These values were generally higher than those reported by Tuzen and Soylak (2007), Mahalakshmi et al. (2012), Ababneh and Al-Momani (2013), Akalın (2018), El-Sayed and Ali et al. (2020), and Al Ghoul et al. (2020), though De Lima et al. (2021) reported aluminum concentrations below detection limits in seven oil-packed samples while others exceeded our values. A statistically significant difference (*P* < 0.05) was observed between aluminum content in oil-packed tuna compared to sauce- and water-packed samples, further emphasizing the influence of packaging medium on elemental accumulation.

### Toxic metal contamination and regulatory compliance

The potentially toxic elements quantified in this study include tin (Sn), mercury (Hg), lead (Pb), and arsenic (As). Additionally, aluminum (Al), although not classified as a heavy metal, was also monitored as a recognised neurotoxic contaminant (see Sect. 4.2). None of these elements has an established beneficial role in human physiology at the concentrations encountered in foodstuffs. Their presence in canned seafood is primarily attributable to environmental pollution, bioaccumulation through aquatic food chains, and migration from processing equipment and packaging materials (WHO 2011; EFSA 2006). Mercury and arsenic are of particular concern in tuna due to their tendency to accumulate in large predatory fish, while tin is primarily associated with migration from tin-plated can linings, and lead reflects general environmental contamination of marine ecosystems.

Mercury contamination represents the most significant food safety concern identified in this investigation. Mean mercury concentrations were measured at 0.416 ± 0.050 mg/kg in sauce-packed, 0.157 ± 0.012 mg/kg in water-packed, and 0.662 ± 0.076 mg/kg in oil-packed samples, with an overall mean of 0.412 ± 0.036 mg/kg across all analyzed samples. Importantly, one sauce-based sample (2.86%) and five oil-based samples (14.29%) exceeded the maximum residue limit of 1.0 mg/kg established by TFC (2011), while one sauce-based (2.86%) and two oil-based samples (5.71%) exceeded the 1.2 mg/kg threshold specified by CAC (2024). These findings indicate that a small but significant proportion (5.71% overall) of canned tuna products available in the Turkish market may pose potential health risks due to elevated mercury levels, with violations predominantly occurring in oil-packed products. The mercury values obtained in this study were comparable to those reported in numerous international investigations (Voegborlo et al. 1999; Khansari et al. 2005; Mol 2010, 2011; Chahid et al. 2015; Ababneh and Al-Momani 2013; Novakov et al. 2017; El-Sayed and Ali 2020; Rodriguez-Mendivil et al. 2019), lower than findings by Mahalakshmi et al. (2012) and Hanis et al. (2024), and higher than results reported by Sadighara et al. (2022), Aziz et al. (2024), and El-Senousi et al. (2025). Mercury, particularly in its methylmercury form, bioaccumulates through aquatic food chains and exhibits high neurotoxicity, with well-documented associations with cognitive impairment, developmental neurotoxicity in children, cardiovascular disease, and immunosuppression, necessitating stringent regulatory surveillance and consumer awareness.

Arsenic contamination was detected at high frequencies across all packaging types. Mean concentrations were 0.807 ± 0.059 mg/kg in sauce-based, 0.651 ± 0.029 mg/kg in water-packed, and 3.018 ± 0.258 mg/kg in oil-packed samples, with an overall mean of 1.493 ± 0.138 mg/kg. The highest arsenic concentration detected was 7.08 mg/kg in oil-packed tuna, while the lowest was 0.31 mg/kg in sauce-based samples. Importantly, 80% of sauce-based, all oil-packed (100%), and 74.29% of water-packed samples exceeded the maximum permissible limit established by FAO/WHO (2015). These elevated levels are particularly concerning given arsenic’s well-established carcinogenic properties and documented associations with cardiovascular complications, skin lesions, and increased risks of lung cancer (Sullivan et al. 1987; Karagas et al. 2004; Marshall et al. 2007; McCarty et al. 2007; Mumford et al. 2007). The arsenic concentrations observed in this study were higher than those reported by Khansari et al. (2005) and Ababneh and Al-Momani (2013), comparable to or lower than findings by Novakov et al. (2017) and Akalın (2018), substantially lower than values documented by De Lima et al. (2021), and contrasted with Hanis et al. (2024) who reported levels below detection limits. It should be emphasized that the present study measured total arsenic, as speciation analysis was not performed. Given that inorganic arsenic is primarily responsible for toxicity while organic arsenic species are generally considered less harmful, the actual health risk associated with arsenic exposure may be overestimated. Nevertheless, the widespread exceedance of regulatory limits, particularly in oil-packed samples, indicates an need for further investigation using speciation-based analytical approaches to accurately assess the proportion of toxic inorganic arsenic species and refine risk assessments.

Lead compliance was satisfactory across all analyzed samples. Mean lead concentrations were measured at 0.097 ± 0.008 mg/kg in sauce-based, 0.060 ± 0.04 mg/kg in water-packed, and 0.109 ± 0.010 mg/kg in oil-packed tuna, with an overall mean of 0.092 ± 0.05 mg/kg. All samples (100%) complied with the maximum residue limit of 0.30 mg/kg established by both TFC (2011) and CAC (2025), indicating no immediate health risk associated with lead exposure from canned tuna consumption in the Turkish market. The lead values determined in this study were generally higher than those reported by the majority of comparative investigations (Khansari et al. 2005; Ashraf et al. 2006; Mahalakshmi et al. 2012; Chahid et al. 2015; Voegborlo et al. 1999; Çelik and Oehlenschlager 2007; Mol 2010, 2011; Iwegbue 2015; Novakov et al. 2017; Rodriguez-Mendivil et al. 2019; El-Sayed and Ali et al. 2020; Sadighara et al. 2022), though still well below regulatory thresholds. Conversely, lower values were observed compared to findings by Tuzen and Soylak (2007) for several canned fish species, Anishchenko et al. (2017) for certain products, Herrera-Herrera et al. (2019), and Akalın (2018), while Jarosz-Krzeminska et al. (2020) and Hanis et al. (2024) reported lead levels below detection limits. Zamand et al. (2024) documented that some canned tuna samples in their study exceeded permitted lead limits. Aziz et al. (2024) and Al-Kazaghly et al. (2025) reported highest lead concentrations in specific tissue types (gills and stomach) of tuna and sardines.

Tin concentrations, while elevated in oil-packed samples, remained well below regulatory limits. Mean values were 39.978 ± 0.527 mg/kg in sauce-based, 37.019 ± 0.474 mg/kg in water-based, and 58.858 ± 3.459 mg/kg in oil-based canned tuna, with an overall mean of 45.091 ± 1.510 mg/kg. All 105 analyzed samples (100%) remained below the maximum residue limit of 200 mg/kg established by TFC (2011) and substantially below the 250 mg/kg threshold reported by EFSA (2006) to cause gastrointestinal effects, indicating no immediate health risk. Tin migration from tin-plated cans into the food matrix is a well-recognized phenomenon in canned food products, and the elevated levels observed in oil-packed samples are consistent with previous reports (Mol 2011; Anishchenko et al. 2017), likely reflecting enhanced metal migration in lipid-based media. The tin values determined in this study were higher than those reported by Mol (2010, 2011), Anishchenko et al. (2017), Al Ghoul et al. (2020), and Sadighara et al. (2022), while Khansari et al. (2005) were unable to detect tin in their canned tuna samples.

### Public health implications and risk assessment

The frequent exceedance of recommended daily intakes for multiple essential elements, coupled with regulatory violations for mercury and widespread arsenic contamination, necessitates careful reconsideration of consumption patterns and public health guidance. While canned tuna unquestionably represents a valuable, convenient, and affordable source of high-quality protein, omega-3 polyunsaturated fatty acids, and essential micronutrients, the cumulative exposure to both essential minerals at excessive levels and potentially toxic metals poses significant health risks that cannot be overlooked.

These risks are particularly pronounced for vulnerable population groups including pregnant women (where methylmercury exposure may cause neurodevelopmental deficits in the fetus; the EPA reference dose for methylmercury is 0.0001 mg/kg body weight/day, while EFSA has established a tolerable weekly intake of 1.3 µg/kg body weight/week for pregnant women and women of childbearing age (EFSA 2006)), young children (whose developing nervous systems are especially susceptible to neurotoxic effects; the WHO provisional tolerable weekly intake for methylmercury is 1.6 µg/kg body weight/week for the general population (WHO 2011)), individuals with compromised renal function (who cannot efficiently eliminate excess minerals and heavy metals), and those with pre-existing cardiovascular conditions (where excessive mineral intake may exacerbate underlying pathology).

Health risk assessment conducted in the present study indicated that arsenic exposure through canned tuna consumption may represent a potential non-carcinogenic risk, particularly for oil-packed products, as reflected by target hazard quotient (THQ) values exceeding unity in certain product categories. Mercury exposure approached the risk threshold established by international regulatory agencies, suggesting that frequent consumption of oil-packed tuna could substantially increase health concerns for vulnerable populations, particularly pregnant women and young children. In contrast, lead exposure remained within acceptable safety limits across all analyzed samples and did not constitute a significant health risk based on current regulatory thresholds. It should be reiterated that total arsenic was measured in this investigation, comprehensive speciation analysis distinguishing between toxic inorganic arsenic species and less harmful organic arsenic compounds would be required to refine these risk estimates and provide more accurate guidance on safe consumption levels.

Current findings strongly suggest that consumption of oil-packed tuna products should be moderated, particularly for individuals in vulnerable population categories. Water-packed tuna may represent a comparatively safer alternative for regular consumption, given its significantly lower concentrations of both essential minerals and toxic metals, though comprehensive risk-benefit analyses incorporating nutritional adequacy considerations are warranted to provide evidence-based dietary recommendations. Consumers should be systematically informed through public health campaigns and product labeling initiatives about the differential metal content across packaging media to enable informed dietary choices and promote safer consumption practices. Regulatory authorities should consider implementing clearer consumption guidelines specifying recommended frequencies for different product types and population groups, similar to existing advisories for large predatory fish species.

### Study limitations and future research directions

Several limitations of the present investigation should be acknowledged. First, although samples were collected from multiple retail channels — including large retail chains, local supermarkets, and online platforms — the study was geographically anchored in Van Province, which may limit the direct generalizability of findings to other Turkish regions with different market compositions. Future studies should therefore expand both the geographic scope and the number of brands analyzed, incorporating a wider range of products available across local and national markets, to provide a more representative assessment of canned tuna safety in Türkiye. Second, temporal variations in metal contamination were not systematically assessed, as all samples were collected during a concentrated three-month period, precluding evaluation of seasonal fluctuations or long-term trends in contamination levels. Third, the study measured total arsenic concentrations without performing speciation analysis to distinguish between highly toxic inorganic arsenic and less harmful organic arsenic species, potentially leading to overestimation of actual health risks. Fourth, metal bioavailability from different packaging media was not experimentally determined, and differences in bioavailability could significantly influence actual dietary exposure and associated health risks.

Future research should prioritize several important areas to address these limitations and extend current knowledge. First, broader sampling campaigns encompassing multiple regions within Türkiye and diverse retail channels — including both physical and online markets — are needed to assess the generalizability of these findings and identify potential geographic or market-based patterns in contamination. Second, longitudinal monitoring programs should be established to evaluate temporal trends in metal contamination and assess the effectiveness of regulatory interventions and industry quality control measures. Third, comprehensive metal speciation studies, particularly for arsenic, are essential to distinguish toxic from non-toxic forms and refine risk assessments. Fourth, in vitro and in vivo bioavailability studies should be conducted to determine the proportion of metals actually absorbed from different packaging media and assess the physiological impact of observed contamination levels. Fifth, systematic investigation of the specific contributions of oils, spices, seasonings, and other additives to overall metal content in various product formulations would provide valuable insights for industry quality control and regulatory oversight. Sixth, examination of the efficacy of different can coating materials, processing equipment specifications, and manufacturing protocols in reducing metal contamination would inform evidence-based recommendations for industry best practices. Finally, comprehensive dietary exposure modeling incorporating actual consumption frequency data from population-based surveys, coupled with integrated risk-benefit assessments that balance nutritional advantages against contamination risks, would provide the empirical foundation for evidence-based public health guidance and regulatory policy development.

### Regulatory and industry recommendations

Based on the findings of this comprehensive investigation, several evidence-based recommendations can be formulated for industry stakeholders, regulatory authorities, and public health agencies. Industry stakeholders should implement systematic pre-production monitoring programs for heavy metals in all raw materials, including fish, processing oils, spices, and other ingredients, excluding inputs with elevated contaminant levels from the manufacturing chain before processing begins. Robust quality control measures should be adopted throughout the production process to minimize potential metal migration from processing equipment, pipes, and packaging materials, including regular equipment maintenance, use of food-grade materials, and installation of appropriate coatings and barriers. The selection of high-quality, uncontaminated oils and spices from verified sources free from industrial waste contamination, air and soil pollution, or proximity to highways and other contamination sources should be prioritized through rigorous supplier qualification and auditing programs.

Regulatory authorities are strongly encouraged to consider the establishment of stricter maximum residue limits for arsenic in canned tuna products, given the widespread exceedance of current FAO/WHO guidelines observed in this study and the well-established carcinogenic properties of inorganic arsenic. Enhanced surveillance and enforcement mechanisms should be implemented to ensure strict compliance with existing mercury regulations, with particular attention to oil-packed products which demonstrated the highest violation rates. Development of comprehensive regulatory frameworks addressing not only toxic metals but also excessive levels of essential elements should be considered, given the documented health risks associated with chronic overconsumption of minerals such as iron, selenium, zinc, and potassium. Mandatory product labeling disclosing packaging medium and encouraging awareness of differential metal content across product types would empower consumers to make informed dietary choices aligned with their individual health status and risk profiles.

Public health agencies should develop and systematically disseminate evidence-based consumer education materials addressing appropriate consumption frequencies for different types of canned tuna products, with population-specific guidance for vulnerable groups including pregnant women, lactating mothers, young children, elderly individuals, and those with chronic kidney disease or cardiovascular conditions. These educational initiatives should clearly communicate both the nutritional benefits of canned tuna consumption (high-quality protein, omega-3 fatty acids, essential minerals) and the potential health risks associated with excessive intake (heavy metal exposure, mineral overload), enabling consumers to optimize the risk-benefit balance through informed consumption choices.

## Conclusions

This comprehensive analysis of 105 canned tuna samples collected from Van Province, eastern Türkiye, revealed substantial and statistically significant variations in mineral and heavy metal content across different packaging media, with oil-packed tuna consistently exhibiting the highest concentrations of both essential minerals (Mg, P, K, Ca, Fe, Cu, Zn, Se) and potentially toxic or non-essential elements (Al, As, Hg, Sn). Although aluminum is not classified as an essential element in human nutrition, its elevated concentrations in oil-packed products — potentially attributable to migration from processing equipment and packaging materials — represent an additional contamination concern warranting further investigation.

Mercury contamination remains a significant food safety concern, with 5.71% of samples exceeding the Turkish Food Codex limit of 1.0 mg/kg and 2.85% exceeding the Codex Alimentarius Commission threshold of 1.2 mg/kg, with regulatory violations predominantly occurring in oil-packed products. Arsenic contamination was detected at high frequencies, with 80% of sauce-packed, all oil-packed (100%), and 74.29% of water-packed samples exceeding the FAO/WHO maximum permissible limit. Although all samples demonstrated compliance with lead and tin regulations, the frequent exceedance of recommended daily intakes for multiple essential elements (Mg, P, K, Fe, Cu, Zn, Se) raises important questions regarding appropriate consumption frequencies and portion sizes, particularly for vulnerable populations including pregnant women, young children, and individuals with compromised renal or cardiovascular function.

The ICP-MS analytical method employed in this investigation, while not constituting a formal method validation study, was rigorously assessed for performance through the use of certified reference material (DORM-4), procedural blanks, matrix spike recovery tests, and duplicate analyses. The method provided reliable, sensitive, and accurate determination of 15 elements simultaneously, with recoveries of 95–102% for all analytes, demonstrating adequate sensitivity and reliability for food safety monitoring and regulatory compliance assessment. These performance characteristics support the suitability of the applied method for routine elemental analysis of canned seafood products.

The findings underscore the important importance of considering packaging medium as a key determinant of elemental composition in canned tuna products, with clear implications for consumer choice, industry quality control, and regulatory oversight.

The results emphasize the need for enhanced quality control throughout the entire canned tuna supply chain, from careful selection and vetting of raw materials through processing, packaging, and distribution. Stricter regulatory enforcement for arsenic, continued vigilant surveillance for mercury, mandatory disclosure of packaging medium on product labels, and comprehensive consumer education regarding the differential metal content across product types are essential measures to ensure food safety and protect public health. Water-packed tuna may represent a comparatively safer alternative for regular consumption based on lower overall metal concentrations, though comprehensive risk-benefit analyses incorporating nutritional adequacy, sensory acceptability, and consumer preferences are warranted to provide holistic dietary guidance.

Future research priorities should include broader geographic sampling campaigns, systematic temporal monitoring to assess trends and evaluate intervention effectiveness, metal speciation studies (particularly for arsenic), bioavailability assessments across different packaging media, and integrated risk-benefit analyses that comprehensively consider both nutritional adequacy and contaminant exposure in the context of realistic consumption patterns. This study provides important baseline data for ongoing food safety monitoring in Türkiye and contributes valuable comparative information to the global scientific understanding of heavy metal contamination in canned seafood products, supporting evidence-based regulatory policy development and public health protection strategies.

## Data Availability

No datasets were generated or analysed during the current study.
